# Impact of Different DOACs on Complications of TBI After Low-Energy Trauma

**DOI:** 10.3390/jcm14248787

**Published:** 2025-12-11

**Authors:** Anna Antoni, Philipp Puhl, Lukas Wedrich, Rebecca Wagner, Matthias Millesi, Valerie Weihs, Elisabeth Schwendenwein, Silke Aldrian, Stefan Hajdu

**Affiliations:** 1 Department of Orthopaedics and Trauma-Surgery, Medical University of Vienna, 1090 Vienna, Austria; 2 Department of Orthopaedics and Traumatology, Klinik Floridsdorf, Vienna Healthcare Group, 1210 Vienna, Austria; 3 Department of Neurosurgery, Medical University of Vienna, 1090 Vienna, Austria

**Keywords:** traumatic brain injury, TBI, mild traumatic brain injury, direct oral anticoagulants, DOACs, antithrombotic therapy, geriatric trauma, silver trauma, low-energy trauma

## Abstract

**Background/Objectives:** While direct oral anticoagulants (DOACs) are widely used, robust evidence for low-energy trauma is scarce. Studies have shown similar or better outcomes of traumatic brain injury (TBI) under DOAC therapy compared to vitamin K antagonists, but there is limited data on the differences among DOAC types. **Methods:** We performed a retrospective study of TBI patients with pre-injury DOACs who presented to our level 1 trauma unit and received cranial computed tomography. Only low-energy trauma mechanisms were included. **Results:** We included 643 patients with an average age of 82 years. As per the Glasgow Coma Scale, 637 patients (99.1%) had a mild TBI and 34 patients (5.3%) had intracranial hematomas. No delayed intracranial bleeding occurred during in-hospital observation. Rivaroxaban was the most frequent DOAC (278, 43.2%), followed by apixaban (221, 34.4%), dabigatran (84, 13.1%), and edoxaban (60, 9.3%). Neurosurgical interventions were performed in three cases (0.5%). The head injury-related in-hospital mortality was 0.9% (six patients). Fisher’s Exact Test and regression analysis did not demonstrate statistically significant differences among the DOAC types regarding occurrence of intracranial bleeding, surgical interventions, or mortality. **Conclusions:** We found no statistically significant differences between DOACs regarding complications of TBI after low-energy trauma. This study shows an overall low risk of complications after low-energy trauma in a predominantly geriatric population with TBI and DOAC therapy.

## 1. Introduction

Injuries from low-energy trauma in a geriatric population are on the rise worldwide due to demographic changes [1,2]. Low-energy trauma is defined as a low-energy impact to the body such as a fall from standing height or less. Low-energy trauma mechanisms often result in minor or no injuries compared to high-energy trauma, such as falls from greater heights or motor vehicle accidents. Despite the lower risk for severe injuries after low-energy trauma, they can result in life-threatening injuries, especially in older adults or those with pre-injury antithrombotic therapy (ATT) or underlying health conditions such as frailty or osteoporosis [2,3].

Traumatic brain injuries (TBIs) are common consequences of low-energy trauma in the elderly population. The majority of these TBIs fall into the spectrum of minor or mild TBI [3,4]. The most common TBI classification is based on the clinical Glasgow Coma Scale (GCS). The literature on mild TBI often lacks clear definitions and distinction of minor and mild TBI. This led to different rates of complications and conclusions in the literature and ongoing research efforts to achieve clearer definitions in the future [5]. Minor TBI or head trauma does not include neurological symptoms, while patients with mild TBI show neurological symptoms such as unconsciousness or amnesia. Due to the large spectrum of severity of minor and mild TBI, this introduces difficulties, especially for patients with cognitive deficits. For these patients, often the neurological symptoms caused by the head trauma cannot safely be differentiated from preexisting conditions or acute delirium.

Age is an independent risk factor for complications and higher morbidity of TBI [6,7]. Despite age, the Physical Status Classification System of the American Society of Anesthesiologists (ASA) has shown that it worsens the outcome after moderate and severe TBI [8].

Various studies and meta-analyses found that any pre-injury antithrombotic therapy (ATT) increases the risk of intracranial bleeding and its complications after TBI [9,10]. Among ATT, vitamin K antagonists (VKAs) showed the highest complications after TBI, including increased mortality [11]. In recent years, DOACs have been investigated and compared to VKAs and antiplatelet therapies (APTs) [12]. For some years, conflicting results were found, but recent meta-analyses showed a lower risk of TBI complications for DOACs compared to VKAs and dual APT [13]. Frequently used DOACs are rivaroxaban, apixaban, edoxaban, and dabigatran. Their safety profile regarding the prevention of thromboembolic events and reduction in complications compared to VKAs has been investigated thoroughly [14]. But when DOACs were introduced to the market and cases of TBI under ATT were on the rise, there was concern among emergency and trauma physicians due to a lack of data for trauma patients and initially a lack of antidotes for DOACs [15,16]. Therefore, some institutions adopted protocols with a high suspicion for intracranial hemorrhages and complications following mild TBI [17,18,19,20,21].

Differences among the types of DOACs were investigated regarding their treatment goal and general complications and it was found that the safety profile of apixaban was higher compared to other DOACs regarding gastrointestinal bleeds [22]. A meta-analysis found that rivaroxaban posed an increased risk of intracranial hemorrhages compared to other DOAC types [23]. Another meta-analysis concluded that the risk of intracranial hemorrhage may differ between DOACs. They found that dabigatran was the safest DOAC, while rivaroxaban ranked last [24].

To our knowledge, there are no large studies which compare the risks of different DOACs for TBI patients. Thus, the aim of the study was to investigate the comparative risk of different types of DOACs regarding the risk of intracranial bleeding, delayed intracranial bleeding, neurosurgical interventions, and mortality in patients with TBI after a low-energy trauma.

## 2. Materials and Methods

We performed a retrospective study of all TBI patients with pre-injury DOAC therapy between January 2017 and August 2018 submitted to an urban level I trauma center. The DOACs were rivaroxaban (Xarelto^®^ Bayer Vital GmbH, Leverkusen, Germany), apixaban (Eliquis^®^ Pfizer Inc., New York, NY, USA), dabigatran (Pradaxa^®^ Boehringer Ingelheim International GmbH, Ingelheim am Rhein, Germany), and edoxaban (Lixiana^®^ Daiichi Sankyo Co., Tokyo, Japan).

Only low-energy trauma mechanisms were included. Any high-energy trauma mechanisms such as a fall from a greater height (including falls on stairs) or motor vehicle accidents were excluded.

TBIs were defined as all head injuries ranging from minor TBI or head trauma (without neurological symptoms), mild TBI (with symptoms, GCS 13–15), moderate TBI (GCS 9–12), to severe TBI (GCS 3–8). Additionally, the severity of the TBI was classified according to the abbreviated injury severity scale (AIS) [25].

The study was approved by the local ethics committee (1905/2021).

At the time of data acquisition, our institution followed a protocol to perform cranial computed tomography (CCT) and admit all TBI patients with pre-injury DOAC therapy for neurological observation for a minimum of 24 h, unless the patient lived in a skilled nursing facility. During in-hospital monitoring, a follow-up CCT was performed only in cases of neurological deterioration. Patients with intracranial hematomas were treated either surgically or conservatively with prolonged in-hospital observation based on the extent of the hematoma and neurological symptoms.

Statistical analysis was performed using SPSS (version 28.0.1.0). Binary logistic regression was used to calculate the odds ratios (ORs) with 95% confidence intervals (CIs) for intracranial hematoma events. To compare the risk of intracranial bleeding associated with different DOACs, separate analyses were performed using rivaroxaban and dabigatran, as rivaroxaban represents the most frequently used DOAC and is described in the literature with the highest risk for intracranial hemorrhages. Dabigatran represents the only direct thrombin inhibitor in the DOAC groups. Comparing these agents against all other DOACs combined allowed us to focus on clinically meaningful contrasts while maintaining a sufficiently large reference group and avoiding model overcomplexity. Separate logistic regression analyses were performed to increase statistical power under conditions of sparse event data.

Given the small number of events, Fisher’s Exact Test was performed to provide a more accurate estimation of statistical significance. A *p*-value of <0.05 was considered statistically significant.

All statistical analyses were performed on an exploratory basis. Due to the low number of intracranial hematoma events, statistical power was limited and the results should be interpreted as hypothesis-generating rather than confirmatory.

## 3. Results

We included 643 patients with TBI after low-energy trauma and pre-injury DOAC therapy. The average age of the patients was 82 years (33–102) and only 3.3% of patients (21) were younger than 60 years. Most patients were female (60.5%, 389).

A diagnosis of pre-injury dementia was documented in 9.0% of patients (58). All patients had at least one pre-existing systemic disease, as shown by the necessity of oral anticoagulation, while 33.6% of patients (216) were multimorbid with at least three systemic diseases.

Rivaroxaban was the most frequent DOAC (278, 43.2%), followed by apixaban (221, 34.4%), dabigatran (84, 13.1%), and edoxaban (60, 9.3%), as shown in Table 1.

Head wounds were present in 74.8% of patients (481) and additional extracranial injuries were documented in 18.0% (116).

As per the Glasgow Coma Scale, 637 patients (99.1%) had a minor or mild TBI. Loss of consciousness or amnesia related to the injury was reported in 11.0% (71).

Intracranial hematomas were detected on the CCT of 34 patients (5.3%). Based on the morphology of the hematoma and threat to the patient’s life, the Abbreviated Injury Score (AIS) was calculated as AIS 1 (minor) in 94.7% (609), AIS 2 (moderate) in 2.0% (13), AIS 3 (serious) 2.0% (13), AIS 4 (severe) in 1.1% (7), AIS 5 (critical) in 0.2% (1), and AIS 6 (maximal) in no case.

No cases of delayed intracranial bleeding were observed during in-hospital monitoring based on follow-up CCTs in case of neurological deterioration.

The average length of stay (LOS) of all admitted patients was 4 days (1–81). Patients without intracranial hematoma had an average LOS of 3 days, while surviving patients with hematomas had an average LOS of 12 days. A total of 0.9% of patients (six) were treated at an intensive care unit (ICU). Patients were discharged from the emergency room to a skilled nursing facility after an unremarkable CCT in 2.3% (15) of cases.

Patients had intracranial hematomas with pre-injury rivaroxaban in 4.7% (13/278), apixaban in 5.9% (13/221), dabigatran in 3.6% (3/84), and edoxaban in 8.3% (5/60) of cases.

Neurosurgical interventions were performed in 0.5% of cases overall (3). One patient each had pre-injury rivaroxaban (0.4%, 1/278), apixaban (0.5%, 1/221), and edoxaban (1.7%, 1/60).

The in-hospital mortality was 2.2% (14), but only six patients who demised had intracranial hematomas (0.9%). Of these patients, rivaroxaban was used in one case (0.4%, 1/278), apixaban in two cases (0.9%, 2/221), dabigatran in two cases (2.4%, 2/84), and edoxaban in one case (1.7%, 1/60).

In the rivaroxaban group, the OR for intracranial bleeding compared to all other DOACs was 0.80 (95% CI 0.40–1.63), indicating a slightly lower risk compared to all other DOACs combined. In the dabigatran group, an OR of 0.63 (95% CI 0.19–2.11) was calculated, suggesting a potentially lower bleeding risk compared to the other DOACs combined. In both comparisons, the differences were not statistically significant (*p* = 0.597 for rivaroxaban and *p* = 0.605 for dabigatran). The wide confidence intervals reflect the small event numbers and indicate limited statistical power.

Furthermore, Fisher’s Exact Test did not demonstrate statistically significant differences among the DOAC types regarding occurrence of intracranial bleeding, surgical interventions, or head injury-related mortality.

## 4. Discussion

We found no statistically significant differences between DOACs regarding complications of TBI after low-energy trauma. This is opposed to previous studies that found differences in complications of DOAC treatment. While rivaroxaban was found to have the highest and dabigatran the lowest complication rates among the DOACs regarding gastrointestinal bleeding and intracranial hemorrhages [22,23,24], we did not find any statistically significant differences among DOACs for low-energy trauma TBI. Overall, neither rivaroxaban nor dabigatran showed a significantly different bleeding risk compared to all other DOACs combined. While both substances demonstrated numerically lower risk, these findings are limited by the small number of bleeding cases.

In line with our results, a recent evidence-based international consensus statement on venous thromboprophylaxis stated that “there is insufficient data to demonstrate superiority for one Factor Xa inhibitor over another as venous thromboembolism (VTE) prophylaxis”, regarding efficacy and the safety profile [26].

This study shows an overall low risk of complications after low-energy trauma in a predominantly geriatric population with TBI and DOAC therapy. Intracranial hemorrhages were present on CCTs in 5.3%. There were no delayed intracranial hemorrhages and only 0.5% of patients had neurosurgical interventions. The head injury-related in-hospital mortality was 0.9% despite an average age of 82 years.

The low risk of TBI complications in our study population could be explained by the protocol of our institution, where all patients with visible or reported signs of head trauma, even without neurological symptoms (minor TBI) receive a CCT and, at the time of the study, in-hospital observation for a minimum of 24 h.

The distinction of minor and mild TBI is a common challenge found in the literature on TBI complications. The most common TBI classification is based on the clinical GCS. Due to the large spectrum of severity of mild TBI, this introduces difficulties, especially for patients with cognitive deficits. Furthermore, many studies do not differentiate between mild TBI with or without pathologies on the CCT. Large-scale scientific efforts are ongoing to solve this challenge [5]. A recent study investigated the clinical consequences of repeat imaging for mild TBI patients with pathologies on CCT. They concluded that “repeat head CT in mild TBI patients with no neurological deterioration is not recommended, even in patients with a higher risk of ICH progression” [27]. Studies like these point out the difficulties clinicians face due to the large spectrum of minor and mild TBI.

Regional differences in health care resources influence clinical decision making. Based on the standards in the Austrian health care system, we believe that the rate of 5.3% intracranial hemorrhages in our study still warrants a prompt CCT for all TBI patients with pre-injury DOAC therapy after a low-energy trauma, regardless of the patient’s clinical presentation. This is in line with international recommendations [28,29,30].

In Austria, patients are frequently admitted for neurological observation despite unremarkable CCTs if they had pre-injury ATT [31]. A previous study on DOAC patients found comparable rates of minor and mild TBI complications, with 0.7% surgical interventions after an unremarkable CCT for high- and low-energy trauma mechanisms. One case of delayed intracranial bleeding was found (0.2%) among TBI patients with DOACs in this study [18]. Within the generally low risk for complications of minor and mild TBI, no significant differences were found between DOAC and VKAs, but a lower risk of complications was found for DOACs compared to APT [13,18,32].

The term silver trauma was introduced to describe the trauma of an elderly patient. It aims to emphasize physiological differences in the elderly [33]. Low-energy trauma has a higher risk of severe injuries for elderly patients and altered physiology could possibly mask severe physical distress. Based on the higher risk of complications after low-energy trauma due to an altered physiology, pre-existing ATT, frailty, or multimorbidity, it has been shown that elderly patients after low-energy trauma mechanisms are frequently victims of undertriage [34,35,36]. Undertriage was defined as lack of trauma-team activation despite an Injury Severity Score ISS > 15 in a recent Scandinavian study. They found that 48% of patients 60 years or older fell into this category [3].

On the other hand, there is a somewhat counter-intuitive risk of harmful overtreatment for elderly patients, driven by a physician’s fear of litigation [37,38]. For older patients, there is a high risk of hospital-associated disability. Loyd et al. found a 30% prevalence of hospital-associated disability in acute care for older patients in their meta-analysis [39]. Delirium is one of the main risks for these patients, with a rate of 14–90% [40,41,42]. Assuming a delirium incidence of 14%, this would have affected 2842 patients who were admitted for monitoring due to minor and mild TBI in Austria in 2023 [43]. These factors require medical decision making based on scientific data and awareness of these risk factors for high-incidence diseases such as TBI after low-energy trauma in the elderly population.

The large European multicenter study CENTER-TBI investigated outcomes for elderly patients and concluded that “there should not be pessimism about outcomes in older adults who survive” [4]. These contrary perspectives add further complexity to the treatment of TBI in elderly patients.

### Limitations

The retrospective design is a limitation of this study. Mild and minor TBI cannot be distinguished in cases where amnesia, unconsciousness, or reduced GCS score are not documented. This results in a potential bias of the study, in such that most of the included patients had only a head injury or minor TBI without neurological symptoms after the injury. But this is a reality for emergency and trauma physicians who assess geriatric patients with a likelihood of underlying cognitive deficits. In such patients, neurological symptoms after the injury cannot safely be excluded. Therefore, any guidelines for the management of elderly patients with TBI should differentiate between minor (head injury without neurological symptoms) and mild TBI (with neurological symptoms).

We did not perform routine follow-up CCTs during observation, only conducting them in cases of neurological deterioration. Therefore, cases of minor delayed intracranial bleeding might have been undiagnosed. However, a previous study showed that most delayed intracranial hemorrhages that were detected by routine follow-up CCT after 24 to 48 h had no therapeutic consequence [17,18]. Furthermore, the low absolute number of intracranial hemorrhage events (5.3%) reduces the statistical power to detect small differences between DOAC types.

## 5. Conclusions

We found no statistically significant differences between DOACs regarding complications of TBI after low-energy trauma. This study shows an overall low risk of complications after low-energy trauma in a predominantly geriatric population with TBI and DOAC therapy. Compared to the literature, our high-volume institution follows a protocol of high suspicion for intracranial lesions with a low threshold for CCT and in-hospital observation for the geriatric population with ATT. Therefore, our study offers valuable data on the outcomes of TBI under DOAC therapy. We recommend a prompt CCT for all TBI patients with pre-injury DOAC therapy after a low-energy trauma, regardless of the patient’s clinical presentation. For patients with an unremarkable CCT and no ongoing TBI symptoms, we recommend discharge from the emergency department to avoid hospital-associated disability of older patients.

## Figures and Tables

**Table 1:** Types of pre-injury direct oral anticoagulants (DOACs) in traumatic brain injury patients after low-energy trauma with rates of intracranial hematomas, neurosurgical interventions, and head injury-related mortality.

DOAC Type	*n* (%)	Intracranial Hematoma (% of DOAC Type)	Neurosurgical Intervention (% of DOAC Type)	Head Injury-Related Mortality (% of DOAC Type)
All DOACs	643	34 (5.3)	3 (0.5)	6 (0.9)
Rivaroxaban	278 (43.2)	13 (4.7)	1 (0.4)	1 (0.4)
Apixaban	221 (34.4)	13 (5.9)	1 (0.5)	2 (0.9)
Dabigatran	84 (13.1)	3 (3.6)	0	2 (2.4)
Edoxaban	60 (9.3)	5 (8.3)	1 (1.7)	1 (1.7)

## Data Availability

Research data are available upon request.

## Abbreviations

TBI	Traumatic brain injury
CCT	Cranial computed tomography
ATT	Antithrombotic therapy
DOACs	Direct oral anticoagulants
APT	Antiplatelet therapy
GCS	Glasgow Coma Scale
